# Lipid bubbles combined with low-intensity ultrasound enhance the intratumoral accumulation and antitumor effect of pegylated liposomal doxorubicin *in vivo*

**DOI:** 10.1080/10717544.2021.1895907

**Published:** 2021-03-09

**Authors:** Inoru Yokoe, Daiki Omata, Johan Unga, Ryo Suzuki, Kazuo Maruyama, Yoshiharu Okamoto, Tomohiro Osaki

**Affiliations:** a Faculty of Agriculture, Joint Department of Veterinary Clinical Medicine, Tottori University, Tottori, Japan; b Faculty of Pharma-Science, Laboratory of Drug and Gene Delivery Research, Teikyo University, Tokyo, Japan; c Faculty of Pharma-Science, Laboratory of Theranostics, Teikyo University, Tokyo, Japan

**Keywords:** Pegylated liposomal doxorubicin, lipid bubbles, low-intensity ultrasound, syngeneic mouse tumor model, tumor treatment

## Abstract

Pegylated liposomal doxorubicin (PLD) is a representative nanomedicine that has improved tumor selectivity and safety profile. However, the therapeutic superiority of PLD over conventional doxorubicin has been reported to be insignificant in clinical medicine. Combination treatment with microbubbles and ultrasound (US) is a promising strategy for enhancing the antitumor effects of chemotherapeutics by improving drug delivery. Recently, several preclinical studies have shown the drug delivery potential of lipid bubbles (LBs), newly developed monolayer microbubbles, in combination with low-intensity US (LIUS). This study aimed to elucidate whether the combined use of LBs and LIUS enhanced the intratumoral accumulation and antitumor effect of PLD in syngeneic mouse tumor models. Contrast-enhanced US imaging using LBs showed a significant decrease in contrast enhancement after LIUS, indicating that LIUS exposure induced the destruction of LBs in the tumor tissue. A quantitative evaluation revealed that the combined use of LBs and LIUS improved the intratumoral accumulation of PLD. Furthermore, tumor growth was inhibited by combined treatment with PLD, LBs, and LIUS. Therefore, the combined use of LBs and LIUS enhanced the antitumor effect of PLD by increasing its accumulation in the tumor tissue. In conclusion, the present study provides important evidence that the combination of LBs and LIUS is an effective method for enhancing the intratumoral delivery and antitumor effect of PLD *in vivo*.

## 1. Introduction

Chemotherapy plays an important role in the therapeutic management of tumor progression, especially in patients with solid tumors that cannot be resected or have already metastasized (Chabner & Roberts, 2005). Conventional chemotherapy using low-molecular-weight drugs (generally < 1000 Da) has two major problems: limited clinical efficacy for solid tumors and severe adverse effects (Minchinton & Tannock, 2006). These problems stem from the inadequate distribution of chemotherapeutics to the tumor tissue and poor tumor selectivity (Chidambaram et al., 2011; Fang et al., 2011). Nanomedicines (e.g. polymer conjugates, micelles, and liposomes of size ranging from 5 to 200 nm) with improved tumor selectivity and biodistribution have been developed (Danhier, 2016). Pegylated liposomal doxorubicin (PLD) was the first approved nanomedicine worldwide in 1995 (Barenholz, 2012); it has an outer phospholipid bilayer modified with polyethylene glycol, inner doxorubicin, and a mean diameter of 80–90 nm (Gabizon et al., 2003). Unlike conventional doxorubicin, PLD has a prolonged half-life in blood owing to its structural features (Gabizon et al., 1993) and tends to accumulate in the tumor tissue (Gabizon et al., 1994; Gabizon et al., 2003). Although PLD reduces the risk of severe adverse effects such as myelosuppression and cardiac toxicity (Soloman & Gabizon, 2008), a recent meta-analysis refuted the therapeutic superiority of PLD over conventional doxorubicin (Petersen et al., 2016).

Recently, the combination of microbubbles (MBs) and ultrasound (US) has gained attention as a promising technique for improved drug delivery, thereby enhancing the therapeutic effect of nanomedicines (Golombek et al., 2018). MBs, commonly used as a contrast agent in ultrasonography, comprise encapsulating shells (made of proteins, lipids, or polymers) and inner gas cores. Under a low-pressure US field, MBs stably contract and expand through a process called oscillation, in response to acoustic waves (De Jong et al., 2000). In contrast, high-pressure US induces large cycles of oscillation leading to inertial cavitation and, ultimately, destruction of MBs (De Jong et al., 2000; Kudo et al., 2009). These phenomena increase the local permeability of the adjacent biomembrane facilitating the transfer of co-injected drugs from microvessels to the extravascular space (Martin & Dayton, 2013; Paefgen et al., 2015). Many *in vivo* studies have suggested that the combination of MBs and low-intensity US (LIUS) can enhance the antitumor effect of conventional chemotherapeutics including doxorubicin (Sonoda et al., 2007; Watanabe et al., 2008; Lu et al., 2011; Ueno et al., 2011; Sorace et al., 2012; Kotopoulis et al., 2017). However, there is little evidence on applying this combination for augmenting the therapeutic benefits of PLD *in vivo* (Zhu et al., 2015; Bush et al., 2020).

Unga et al. developed new lipid-based MBs termed lipid bubbles (LBs), which comprise an outer phospholipid monolayer and inner perfluoropropane gas core with a mean particle size of approximately 2.7 μm (Unga et al., 2019). Previous *in vivo* studies demonstrated that the combination of LBs and LIUS could induce the delivery of high-molecular-weight agents (Omata et al., 2019; Unga et al., 2020). Therefore, we hypothesized that the combination of LBs and LIUS could improve the delivery of PLD into solid tumors and lead to a strong therapeutic effect. Most recently, we performed a preclinical pilot study on three dogs with spontaneously occurring solid tumors to explore the therapeutic efficacy of the combination treatment using PLD, LBs, and LIUS (Yokoe et al., 2020). The dogs presented a remarkable reduction in tumor volume making combination treatment a potential candidate for solid tumor treatment.

To the best of our knowledge, the underlying mechanism of the combination treatment using PLD, LBs, and LIUS remains unclear. This study aimed to evaluate the influence of the combined use of LBs and LIUS on the intratumoral delivery of PLD and to determine whether the combination could improve the antitumor effect of PLD *in vivo*.

## 2. Materials and methods

### 2.1. Ethics statement

To ensure the welfare of animals used in this study, the experimental protocols involving animals were prepared based on the 3Rs (replacement, reduction, and refinement). Experimental animal use and procedures were approved by the Animal Research Committee of Tottori University (project number: 18-T-47). *In vivo* experiments were performed according to the Institute of Laboratory Animal Resources guidelines for the use of experimental animals.

### 2.2. Preparation of LBs

1,2-Distearoyl-sn-glycero-3-phosphocholine (DSPC), 1,2-distearoyl-sn-glycero-3-phosphoglycerol (DSPG), and N-(carbonyl-methoxypolyethyleneglycol 2000)-1,2-distearoyl-sn-glycero-3-phosphoethanolamine (DSPE-PEG2000) were purchased from NOF Corporation (Tokyo, Japan). Perfluoropropane was purchased from Takachiho Chemical Industrial Co., Ltd. (Tokyo, Japan). As previously reported (Unga et al., 2020), LBs were prepared by homogenization of a lipid dispersion of DSPC, DSPG, and DSPE-PEG2000 in a perfluoropropane atmosphere followed by freeze-drying. Liposomes comprising DSPC, DSPG, and DSPE-PEG2000 in the molar ratio of 30:60:10 were prepared using the lipid film hydration method; 300 μmol of all lipids were mixed with a mixture of chloroform, methanol, aqueous ammonium solution, and Milli-Q water (65:35:4:4, v/v/v/v, respectively). The lipid film was prepared in a rotary evaporator (Eyela N-1100, Tokyo Rikakikai Co. Ltd., Tokyo, Japan) and dried overnight in a vacuum desiccator (ULVAC GCD-136X, ULVAC Inc., Kanagawa, Japan) to completely remove the solvents. The lipid film was then hydrated with 75 mL of 100 mM phosphate buffer (pH 7.4) for 30 min at 65 °C under shaking. The resulting liposomes were sonicated in a bath-type sonicator (Bransonic 2150j-DTH, Branson Ultrasound Co., Danbury, CT, USA) for 10 min. A homogenizer (Labolution MarkII 2.5, Primix Corporation, Hyogo, Japan) was inserted into a mixing vessel containing 75 mL of liposomes and 225 mL of 100 mM phosphate buffer. The air in the vessel was replaced with perfluoropropane. The liposomes were homogenized at 7500 rpm for 60 min at 40 °C. The microbubble dispersion was mixed with an 18% sucrose solution in a 1:1 (v/v) ratio, and 2 mL of the mixture was dispensed into a 5-mL vial. The air in the headspace was replaced with perfluoropropane. The vials were closed with rubber lids and frozen at −30 °C overnight. After freezing, the rubber lid was opened and the vials were freeze-dried at −30 °C for 1 h, −20 °C for 72 h, and 20 °C for 48 h, in a shelf-temperature-controlled drying chamber (Eyela FDU-1100 and Eyela DRC-1100, Tokyo Rikakikai Co. Ltd., Tokyo, Japan). After the drying process was completed, the chamber was filled with perfluoropropane. The vials were then closed with rubber lids and capped with an aluminum cap. Before administration to mice, the freeze-dried LBs were reconstituted in 2 mL of distilled water and shaken gently. The number of LBs was measured using a Multisizer 3 particle counter (Beckman Coulter Inc., Brea, CA, USA). Samples for measuring were prepared by suspending 10 µL of LBs in approximately 50 mL of ISOTON II (Beckman Coulter Inc., Brea, CA, USA); 50 µL of the mixture was quantified. The concentration of LBs was approximately 1.6 × 10^9^ particles/mL. LBs were administered at a dose of approximately 3.2 × 10^7^ particles/mouse (0.02 mL/mouse) in all of the *in vivo* experiments.

### 2.3. Preparation of tumor cells and mouse models

Mouse mammary tumor EMT6 cells were provided by Prof. Yoshihiro Uto (Tokushima University, Japan). EMT6 cells were grown in RPMI-1640 medium (Thermo Fisher Scientific, Inc., Waltham, MA, USA) supplemented with antibiotics (5 mg/mL penicillin, 5 mg/mL streptomycin, and 10 mg/mL neomycin from Thermo Fisher Scientific, Inc., Waltham, MA, USA) and 10% heat-inactivated fetal bovine serum (Nichirei Bioscience Inc., Tokyo, Japan). The cells were maintained at 37 °C in a chamber containing 5% CO_2_.

Six-week-old female BALB/c mice were obtained from Clea Japan, Inc. (Tokyo, Japan). The mice were maintained under conventional conditions. Standard pellet food and water were provided *ad libitum*. EMT6 cells were inoculated subcutaneously in the shaved lower dorsum of the mice at a density of 1 × 10^6^ cells in 0.1 mL of phosphate-buffered saline (PBS, Thermo Fisher Scientific, Inc., Waltham, MA, USA) per mouse.

### 2.4. Diagnostic and therapeutic US devices and their settings

A US imaging device (Arietta60, Hitachi Aloka Medical, Ltd., Tokyo, Japan) with a 2–12 MHz broadband linear probe was used for visualizing contrast-enhanced US images, as described in Section 2.5. In the contrast harmonic imaging mode, the mechanical index was set at 0.22 and the background gain at 60 dB. A focal zone was placed in the deepest area of the scanning window to minimize the destruction of LBs. Every scan was recorded in the local storage of the device. An LIUS generator (UST-770, ITO Co., Ltd., Tokyo, Japan) along with a US transducer, which is a circular disk of 15 mm diameter, was used to provide acoustic effects to LBs in all the experiments. The output settings for US in all the experiments were as follows: frequency, 1 MHz; power intensity, 2 W/cm^2^; duty cycle, 50%; pulse repetition frequency, 100 Hz; and exposure time, 60 s. Before every US application to tumor tissues, a coupling gel (Sonojelly M, Canon Medical Supply Co., Ltd., Tokyo, Japan) was applied adequately to the surface of tumor tissues.

### 2.5. Influence of LIUS on the in vivo stability of LBs

To evaluate the US contrast effect of LBs in the tumor tissue *in vivo*, contrast-enhanced ultrasonography of the tumor tissue was performed in model mice. In addition, the contrast-enhancing effect of LBs before and after LIUS exposure was examined to evaluate the influence of LIUS exposure on the stability of LBs. The experiments were performed when the tumors in the model mice reached approximately 10 mm in length. The mice were randomly divided into two groups of six mice each: LBs (contrast-enhanced US imaging using LBs alone) and LBs + LIUS (exposed to LIUS during contrast-enhanced US imaging using LBs). The mice were anesthetized using an intraperitoneal injection of a mixture of 0.75 mg/kg medetomidine (Dorbene^®^ vet, Kyoritsu Seiyaku Co., Tokyo, Japan), 4 mg/kg midazolam (Dormicum^®^ injection 10 mg, Astellas Pharma Inc., Tokyo, Japan), and 5 mg/kg butorphanol (Vetorphale^®^, Meiji Seika Pharma Co., Ltd., Tokyo, Japan) in saline (dosage = 0.01 mL/g body weight). The mice were placed in the prone position on a warm water bed at 37 °C, and the US probe of the US imaging device was set at the surface of the tumor tissue. Then, LBs were injected through the tail vein, and US imaging was initiated (time point = 0 s). In the LBs group, US imaging was continued without suspension. In the LBs + LIUS group, the US probe was removed, and the scanning was suspended 30 s after the injection of LBs. Immediately, a transducer of the LIUS device was set at the surface of the tumor tissue. Then, the tumor tissue was exposed to LIUS for 60 s. After exposure to LIUS, the US probe of the imaging device was set at the same position again, and the scanning was reinitiated. Scanning was continued for 900 s after the injection of LBs. Individual US images were obtained at 0, 10, 20, 30, 60, 90, 120, 180, 240, 300, 420, 600, and 900 s by taking screenshots from the scanning video. In individual US images, a region of interest was manually set to focus exclusively on the tumor tissues. Mean grayscale intensity (MGI) in the region of interest was calculated depending on the degree of the white signal within the range of 0–255 using image analysis software (ImageJ, National Institutes of Health, Bethesda, MD, USA, http://rsb.info.nih.gov/ij/).

### 2.6. Temperature measurements in the tumor tissues

The temperature in the tumor tissues was measured to evaluate the heating effect caused by the intravenous injection of LBs under LIUS exposure. The experiment was initiated when the tumors in the model mice reached approximately 10 mm in length. Mice were randomly divided into four groups of five mice each: control (untreated), LBs (injected with LBs alone), LIUS (exposed to LIUS alone), and LBs + LIUS (injected with LBs and exposed to LIUS). The mice were anesthetized as described in Section 2.5 and placed in the prone position on a warm water bed at 37 °C. A data logger thermometer (CENTER521, Satotech Inc., Kanagawa, Japan) with a standard temperature sensor was used for the measurements. The sensor was inserted into the center of the tumor tissue. In the LBs and LBs + LIUS groups, LBs were injected through the tail vein. In the LBs + LIUS group, the tumor tissues were exposed to LIUS for 60 s immediately after the injection of LBs. In the LIUS group, the tumor tissues were exposed to LIUS without the injection of LBs. When the LIUS exposure was over, the coupling gel applied on the tumors was gently wiped off with clean cotton. The time of injection of LBs was defined as 0 s. The temperatures of the tumor tissues were monitored continuously from 0 to 80 s.

### 2.7. Influence of LBs and LIUS on the in vitro stability of PLD

*In vitro* stability of PLD was evaluated to check whether the combined use of LBs and LIUS induced the destruction of the liposomal structure of PLD. PLD (Doxil, Janssen Pharmaceutical K.K., Tokyo, Japan) was distributed to 48-well microplates (Falcon 353078, Corning Inc., NY, USA) at 20 μg/well or 200 μg/well. LBs were added to the wells at 3.2 × 10^7^ particles/well, and then PBS was added to all the wells up to 1.5 mL. The transducer of the LIUS device was set at the liquid surface, followed by exposure to LIUS for 60 s. The samples were transferred to 2-mL tubes. Each well was washed with 500 μL of PBS, and the solutions were added to the tubes. The tubes were then weighed and ultracentrifuged for 1 h at approximately 290,000 × *g* at 4 °C. After ultracentrifugation, 1.6 mL of the supernatant was transferred to new 2.0-mL tubes. The tubes with precipitates were weighed, and content fluid was re-suspended. The suspensions were then transferred to new 2.0-mL tubes. TritonX-100 was added to the tubes at a final concentration of 1% to decompose the liposomal structure of PLD. To quantitatively evaluate the weight of doxorubicin in the supernatant and the precipitant, the fluorescence intensity of the samples was measured at excitation and emission wavelengths of 485 and 590 nm, respectively, using a fluorescence microplate reader (PowerScan HT, DS Pharma Biomedical Co., Ltd., Osaka, Japan).

### 2.8. Quantitative evaluation of the PLD delivery into tumor tissues

To quantitatively evaluate the delivery of PLD by the combination of LBs and LIUS, the content of doxorubicin in the tumor tissues was calculated. The experiment was initiated when the tumors in the model mice reached approximately 10 mm in length. PLD was administered at a dose of 10 mg/kg. Mice were randomly divided into three groups of eight or nine mice each: PLD (injected with PLD alone), PLD + LIUS (exposed to LIUS after PLD injection), and PLD + LBs + LIUS (exposed to LIUS after injection of PLD and LBs mixture). The mice were placed in the prone position using a restrainer and without anesthesia. In the PLD and PLD + LIUS groups, PLD was injected through the tail vein. In the PLD + LIUS group, the tumor tissues were exposed to LIUS immediately after the injection of PLD. In the PLD + LBs + LIUS group, a mixture of PLD and LBs was injected through the tail vein, followed by exposure of the tumor tissues to LIUS. The mice were kept in the dark for 1 h. The mice were euthanized by cervical dislocation under deep anesthesia with 5% isoflurane, and the tumor was resected. The excised tumors were weighed and stored at −20 °C until use. The frozen tumors were homogenized using a bead beater-type homogenizer (μT-12, Taitec Co., Saitama, Japan) at 3200 rpm for 2 min in 1 mL of cold PBS. The samples were then centrifuged for 20 min at 17,000 × *g* at 4 °C. After centrifugation, 100 μL of the supernatant was transferred to 96-well black microplates (BD Bioscience, Bedford, USA). Fluorescence intensity of the supernatant was measured at excitation and emission wavelengths of 490 and 590 nm, respectively, using a fluorescence microplate reader (SH-9000lab, Corona Electric, Ibaraki, Japan).

### 2.9. Qualitative evaluation using immunofluorescence staining

Immunofluorescence staining was performed to qualitatively evaluate the intratumoral delivery of macromolecular agents. The experiment was initiated when the tumors in the model mice reached approximately 10 mm in length. Fluorescein isothiocyanate-labeled dextran (FITC-dextran), with a molecular weight of 70,000 Da (Thermo Fisher Scientific, Inc., Tokyo, Japan), was used as a tracer at a dose of 2 mg/mouse. Mice were injected with FITC-dextran and exposed to LIUS after the injection of FITC-dextran or exposed to LIUS after the injection of a mixture of FITC-dextran and LBs (FITC-dextran, FITC-dextran + LIUS, and FITC-dextran + LBs + LIUS groups, respectively). The mice were injected with LBs and FITC-dextran, exposed to LIUS, and isolated in the dark sequentially as described in Section 2.8. After 1 h of isolation in the dark, the mice were euthanized by cervical dislocation under deep anesthesia with 5% isoflurane. The tumor tissues were excised and immersed in 4% paraformaldehyde for 24 h at room temperature (RT), followed by incubation in 20% sucrose (in PBS) overnight at 4 °C. The tumor tissues were then embedded in an optimal cutting temperature compound (Sakura Finetechnical Co., Ltd., Tokyo, Japan) and snap-frozen in acetone with dry ice. Sagittal 30-μm sections were prepared using a cryostat and blocked using 0.1% Tween-20 in a commercial blocking buffer (abcam ab126587, Abcam, Tokyo, Japan)-PBS solution for 1 h at RT. The sections were incubated with rat anti-mouse CD31 antibody (ab7388, 1:500 dilution, Abcam, Tokyo, Japan) in 0.1% Tween-20/blocking buffer solution overnight at 4 °C. The sections were then stained with Alexa Fluor 594-labeled goat anti-rat antibody (ab150160, Abcam, Tokyo, Japan) in 0.1% Tween-20/blocking buffer solution (1:1000 dilution) for 2 h at RT. After mounting with VETASHIELD Vibrance with 4′-6-diamidino-2-phenylindole (DAPI) (Vector Laboratories, Inc., Burlingame, CA), digital images were obtained using an all-in-one microscope (BZ-X810, Keyence, Osaka, Japan). The consecutive 30-μm sections were stained with hematoxylin and eosin (H&E) for histological observation.

### 2.10. Tumor growth inhibition test

Tumor mice models were randomly divided into seven groups: (1) control (untreated); (2) LBs (injected with LBs); (3) LIUS (exposed to LIUS alone); (4) LBs + LIUS (injected with LBs and exposed to LIUS); (5) PLD (injected with PLD alone); (6) PLD + LIUS (injected with PLD and exposed to LIUS); and (7) PLD + LBs + LIUS (the combined treatment of PLD, LBs, and LIUS exposure). The experiment was initiated when the tumors in the model mice reached approximately 5–7 mm in length. The dose of PLD was set at 1 mg/kg. The mice were injected with LBs and/or PLD through the tail vein in a restrainer without anesthesia, except for Groups (1) and (3). The tumor tissues were exposed to LIUS without any drug injection in Group (3) or immediately after drug injection in Groups (4), (6), and (7). The treatment was repeated on 3 days: days 0, 2, and 4. The mice were observed and weighed every other day for 22 days. The tumor size was measured with a digital caliper, and tumor volume was estimated using the formula ‘Length × Width × Thickness × 3.14/6.’ The mice whose inoculated tumors became non-palpable lesions in response to the treatments were evaluated as having complete response (CR). The humane endpoint of the experiment was set as follows: weight loss of >10% in 2 days, total weight loss of >20%, or tumor diameter of >17 mm.

### 2.11. Histological analysis and apoptosis detection

As described in Section 2.10., the treatments were performed on 3 days: days 0, 2, and 4. On day 8, the tumor tissues were excised and fixed in 10% buffered formalin. The samples were embedded in paraffin and sectioned into 4-μm-thick slices. To evaluate the anticancer effect histologically, the sections were stained with H&E. In addition, terminal deoxynucleotidyl transferase-mediated dUTP nick-end labeling (TUNEL) was performed to detect apoptotic cells in consecutive sections using an *in situ* apoptosis detection kit (Takara Bio., Inc., Shiga, Japan) following the manufacturer’s instructions. Digital images were obtained using a BZ-X810 microscope.

### 2.12. Statistical analyses

The results for all parameters are expressed as mean ± standard deviation. The differences in the MGI values at 90 s between the LBs and LBs + LIUS groups were analyzed using the Student’s *t*-test. The differences between the tumor temperatures at 0 and 60 s in each group were analyzed using the paired *t*-test. The differences in the tumor temperatures at 60 s between all the groups were analyzed using Tukey’s multiple comparison test. Data from the *in vitro* experiment on the stability of PLD were analyzed using Tukey’s multiple comparison test. Data from the quantitative evaluation of PLD delivery and tumor growth inhibition tests were analyzed using Tukey’s multiple comparison test. All statistical analyses were performed using a computer software program (XLSTAT, Addinsoft, Paris, France). A *p*-value of <.05 was considered statistically significant for all the tests.

## 3. Results

### 3.1. Influence of LIUS on the in vivo stability of LBs

**Figure 1.**
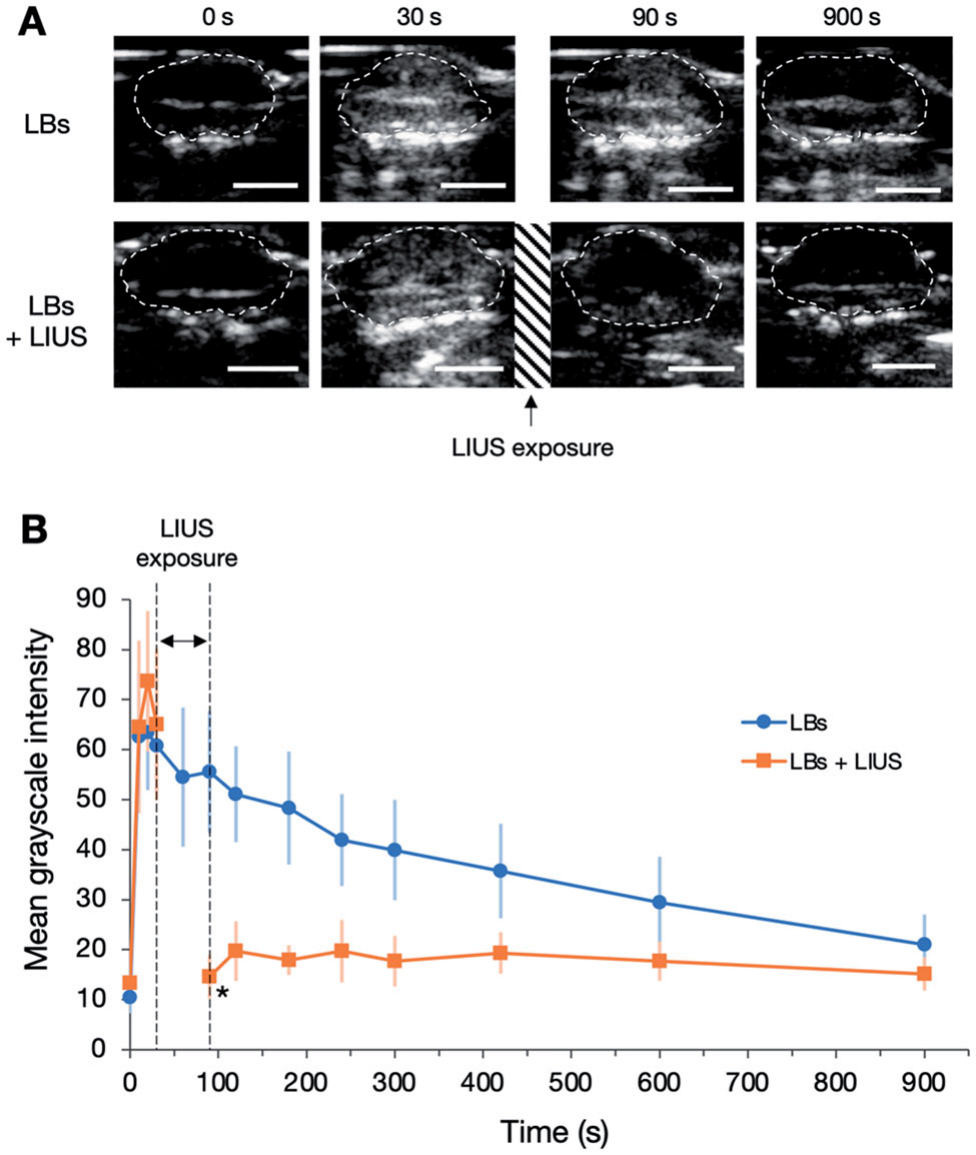
Influence of low-intensity ultrasound (LIUS) on the stability of lipid bubbles (LBs) in the tumor tissue. (A) LBs were administered to mice through the tail vein, and contrast-enhanced images of the tumor tissues were visualized using an ultrasound imaging device for 900 s. The areas within the dashed lines indicate the tumor tissues. In the LBs + LIUS group, contrast-enhanced imaging was suspended between 30 and 90 s, and the tumor tissue was exposed to LIUS during that period. Scale bar: 5 mm. (B) Time-mean grayscale intensity (MGI) curves of the whole-tumor tissues in contrast-enhanced ultrasound imaging. MGI indicates the average degree of white signal in the tumor tissues and was calculated within the range of 0–255. Data are shown as mean ± standard deviation (*n* = 6). *p < .001 for the comparison between LBs and LBs + LIUs at 90 s, using Student’s *t*-test.

Representative US images of tumor tissues are shown in Figure 1(A). In the LBs group, the tumor tissues of mice showed contrast enhancement within several seconds after the injection of LBs. The enhancement of the tumor tissues gradually faded during the observation period. In the LBs + LIUS group, the tumor tissues were exposed to LIUS from 30 to 90 s after the injection of LBs. At 90 s, the contrast enhancement of the tumor tissues declined. The time-MGI curves of the two groups are shown in Figure 1(B). In the LBs group, the MGI of the tumor tissues peaked at 20 s (MGI: 63.5 ± 11.5) after the injection of LBs and then gradually decreased. Similar to the LBs group, in the LBs + LIUS group, the MGI increased immediately after the injection of LBs and peaked at 20 s (MGI: 73.7 ± 14.0). At 90 s after LIUS exposure, the MGI significantly decreased compared with that of the LBs group (14.7 ± 4.8 vs. 55.5 ± 13.4, *p* < .001). The MGI after LIUS exposure remained low during the rest of the observation period.

### 3.2. Temperature measurements in the tumor tissues

**Figure 2.**
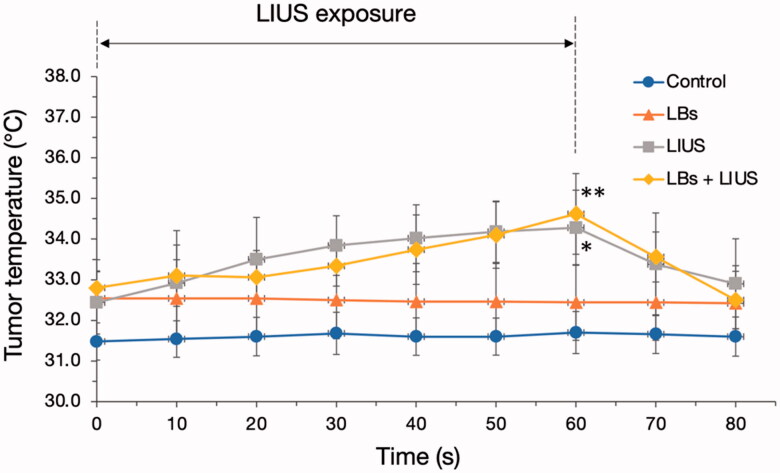
Tumor temperature measurements during and after low-intensity ultrasound (LIUS) exposure. Lipid bubbles (LBs) were injected into the mice through the tail vein, followed by exposure of the tumor tissue to LIUS for 60 s. The tumor temperatures have been plotted every 10 s for 80 s. Data are shown as mean ± standard deviation (*n* = 5). *p = .035 for the temperature difference in the LIUS group between 0 and 60 s and **p = .013 for the temperature difference in the LBs + LIUS group between 0 and 60 s, analyzed using paired *t*-test.

Changes in tumor temperatures in each group are shown in Figure 2. The control and LBs groups did not show any change in tumor temperatures. The tumor temperatures were gradually and significantly increased from 0 to 60 s in the LIUS and LBs + LIUS groups (from 32.4 ± 0.8 to 34.3 ± 0.9 °C, *p* = .035 and from 32.8 ± 0.4 to 34.6 ± 1.0 °C, *p* = .013, respectively), and rapidly decreased after that. The tumor temperatures at 60 s in the two groups were significantly higher than those in the control group (34.3 ± 0.9 vs. 31.7 ± 0.5 °C [LIUS vs. control], *p* = .003; 34.6 ± 1.0 vs. 31.7 ± 0.5 °C [LBs + LIUS vs. control], *p* = .001, respectively). In contrast, there was no significant difference in the tumor temperatures at 60 s between the LIUS and LBs + LIUS groups (34.3 ± 0.9 vs. 34.6 ± 1.0 °C, *p* = .943).

### 3.3. Influence of LBs and LIUS on the in vitro stability of PLD

**Figure 3.**
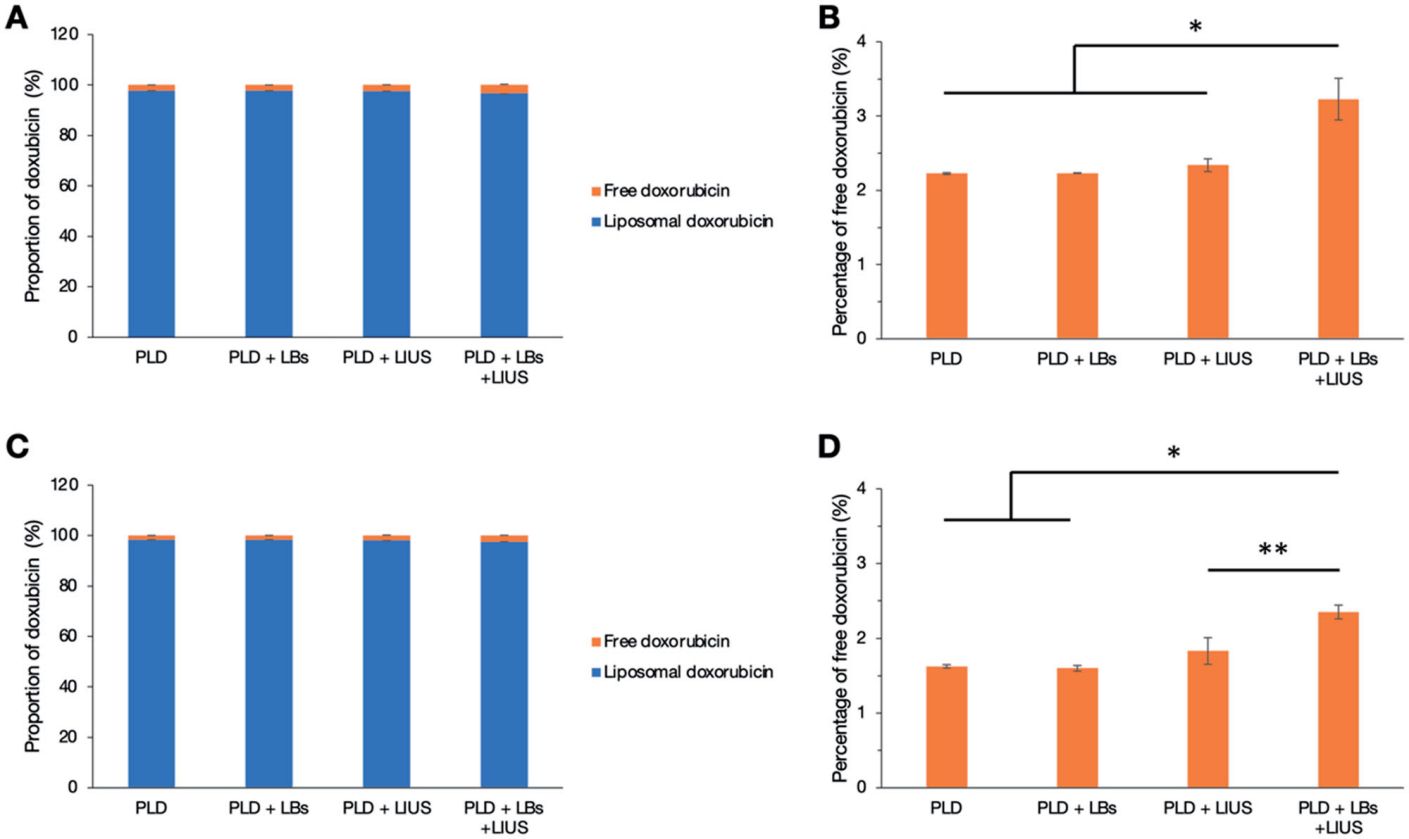
Influence of the combined use of lipid bubbles (LBs) and low-intensity ultrasound (LIUS) on the stability of pegylated liposomal doxorubicin (PLD). PLD and LBs were distributed to 48-well microplates, followed by exposure to LIUS. After ultracentrifugation, fluorescence intensities of the supernatant and the precipitant were measured at excitation and emission wavelengths of 485 and 590 nm (*n* = 3), respectively. (A) The proportion of the doxorubicin weight at a dose of 20 μg/well is shown using a bar graph. (B) The percentage of free doxorubicin at a dose of 20 μg/well is shown. *p < .001 analyzed with Tukey’s multiple comparison test. (C) The proportion of the doxorubicin weight at a dose of 200 μg/well is shown using a bar graph. (D) The percentage of free doxorubicin at the dose of 200 μg/well is shown. *p < .001, **p = .0011 analyzed with Tukey’s multiple comparison test. Error bars with a positive direction denote the standard deviation (SD) of free doxorubicin, and those with a negative direction denote the SD of liposomal doxorubicin in (A) and (C).

Figure 3 shows the proportion of free doxorubicin and doxorubicin extracted from PLD after each treatment using LBs and LIUS. In Figure 3(A,B), doxorubicin was used at a dose of 20 μg/well. In Figure 3(C,D), doxorubicin was used at a tenfold dose of 200 μg/well. The proportion of free doxorubicin was low (less than 4%) in all the groups at both doses (Figure 3(A,C)), although the percentage of free doxorubicin in the PLD + LBs + LIUS group was significantly higher than that in the PLD group at both doses (Figure 3(B,D)).

### 3.4. Quantitative evaluation of the PLD delivery into tumor tissues

**Figure 4.**
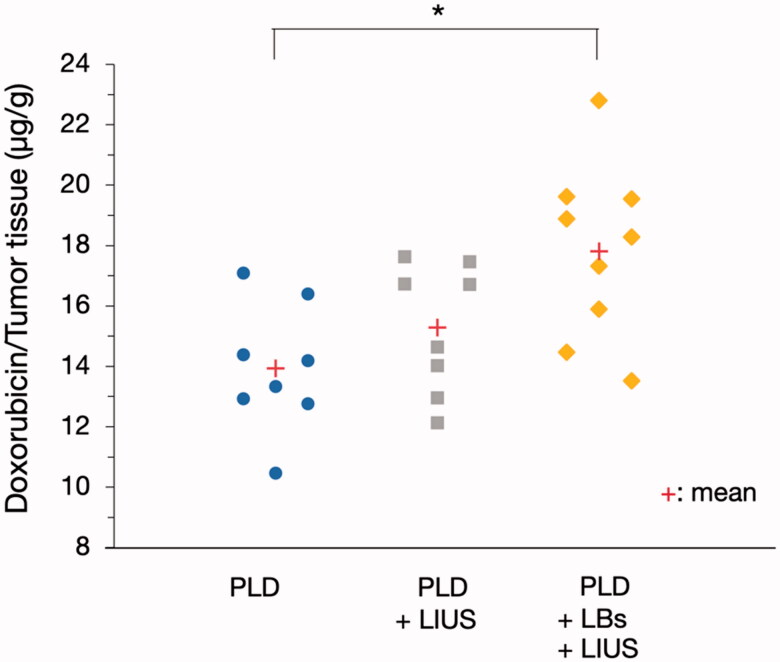
Effect of the combination treatment of lipid bubbles (LBs) and low-intensity ultrasound (LIUS) on the delivery of pegylated liposomal doxorubicin (PLD) to the tumor tissues. A mixture of PLD and LBs was injected through the tail vein, and then, the tumor was exposed to LIUS. After 1 h, the tumor was removed and homogenized for fluorescence intensity measurements in the supernatant at excitation and emission wavelengths of 490 and 590 nm (*n* = 8, 9), respectively. *p = .009, analyzed using Tukey’s multiple comparison test.

The doxorubicin content of the tumor tissues in each group is shown in Figure 4. The doxorubicin content in the PLD + LBs + LIUS group was 1.28 times higher than that in the PLD group (17.8 ± 2.9 vs. 13.9 ± 2.1 μg/g wet tumor tissue, *p* = .009). There was no significant difference in the doxorubicin content between the PLD and PLD + LIUS groups (13.9 ± 2.1 vs. 15.3 ± 2.1 μg/g wet tumor tissue, *p* = .518).

### 3.5. Qualitative evaluation using immunofluorescence staining

**Figure 5.**
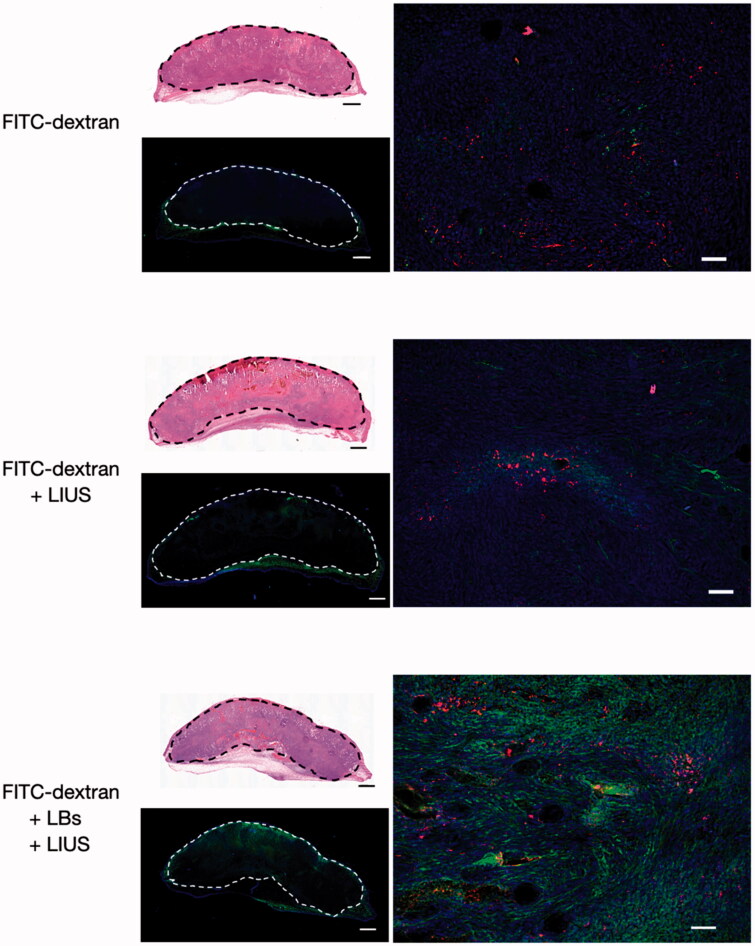
Fluorescent images of the tumor tissues treated with lipid bubbles (LBs) and low-intensity ultrasound (LIUS). A mixture of fluorescein isothiocyanate-labeled dextran (FITC-dextran) and LBs was injected through the tail vein, and the tumor tissue was immediately exposed to LIUS. After 1 h, the tumor was excised and fixed in paraformaldehyde. Consecutive 30-μm cryosections were prepared for immunofluorescence analysis and staining with hematoxylin and eosin. Whole-tumor images were obtained by combining multiple low-magnification images using a BZ-X810 microscope. The areas within the dashed lines indicate the tumor tissues. Green: FITC-dextran, red: CD31 antigen (indicating tumor endothelial cells), and blue: nuclei stained with 4′-6-diamidino-2-phenylindole (DAPI) in the fluorescent images. Scale bar: 1 mm in whole-tumor images and 50 μm in high-magnification images.

Figure 5 shows the microscopic images of the tumor tissues in the FITC-dextran-, FITC-dextran + LIUS-, and FITC-dextran + LBs + LIUS-treated mice. As shown in the low-magnification images, FITC-dextran was mainly observed in the subcutaneous tissue around the tumor in each mouse. FITC-dextran was poorly distributed in the tumor tissues of FITC-dextran and FITC-dextran + LIUS mice, while it was well distributed in the FITC-dextran + LBs + LIUS mice. In the high-magnification fluorescent images, FITC-dextran was sparsely distributed around the tumor vessels in the FITC-dextran and FITC-dextran + LIUS mice. In contrast, FITC-dextran was widely dispersed around the tumor vessels in the FITC-dextran + LBs + LIUS mice.

### 3.6. Tumor growth inhibition test

**Figure 6.**
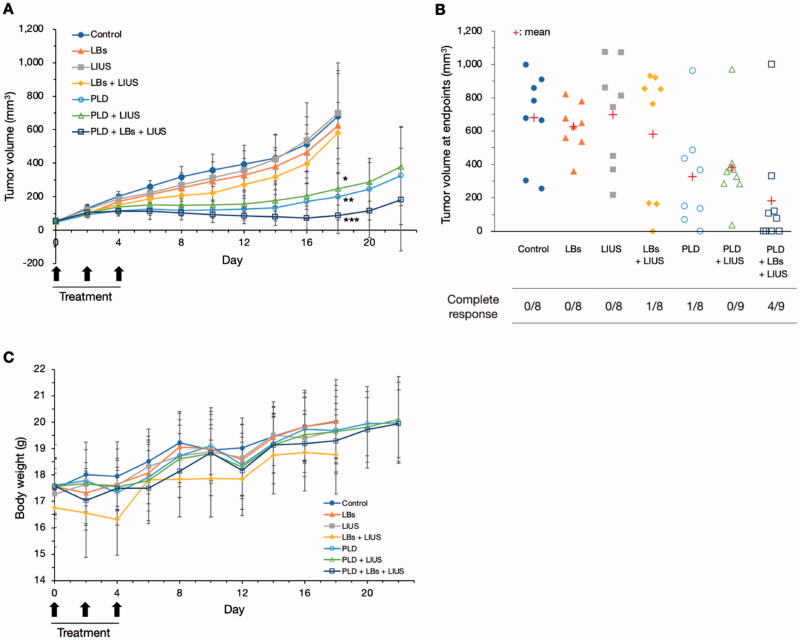
Tumor growth inhibition test using combination treatment with pegylated liposomal doxorubicin (PLD), lipid bubbles (LBs), and low-intensity ultrasound (LIUS) in tumor mice models. A mixture of PLD and LBs was injected through the tail vein, and the tumor tissue was immediately exposed to LIUS. The treatment was repeated on 3 days: days 0, 2, and 4. (A) Changes in tumor volume in each group. Data are shown as means ± standard deviations (*n* = 8, 9). *p = .007, **p = .003, and ***p < .001 for tumor volumes in the PLD, PLD + LIUS, and PLD + LBs + LIUS groups, respectively, compared with that in the control group on day 18, analyzed using Tukey’s multiple comparison test. **(**B) Tumor volume at endpoints in every mouse in each group and the number of mice showing complete responses. **(**C) Changes in body weight during the observation period.

Changes in tumor volume in each group are shown in Figure 6(A). In the control, LBs, LIUS, and LBs + LIUS groups, the tumor volume gradually increased and reached 680.6 ± 255.1, 626.2 ± 136.1, 699.8 ± 300.8, and 581.5 ± 371.6 mm^3^ on day 18, respectively. There were no significant differences in tumor volume between the four groups. The observation was terminated on day 18 in the four groups because the tumor exceeded 17 mm in length (maximum length) in at least one mouse in each group. In the PLD, PLD + LIUS, and PLD + LBs + LIUS groups, the tumor reached 198.9 ± 138.2, 247.2 ± 98.1, and 87.3 ± 107.1 mm^3^, respectively; day 18 measurements confirmed that the growth of tumors in these groups was significantly inhibited compared to the control group (*p* = .003, .007, and <.001, respectively). The three groups were observed until day 22. The tumor volumes in the three groups (PLD, PLD + LIUS, and PLD + LBs + LIUS) on day 22 reached 326.4 ± 292.4, 380.3 ± 233.2, and 182.2 ± 306.4 mm^3^, respectively; there were no significant differences between the three groups. Figure 6(B) shows the tumor volume at the endpoints in every mouse in each group. Compared with the control, LBs, LIUS, and PLD + LIUS groups with no mice showing a CR, the LBs + LIUS and PLD + LIUS groups each had one mouse showing a CR. In contrast, four out of nine mice acquired a CR in the PLD + LBs + LIUS group. The average bodyweight of the mice varied from 16.3 to 20.1 g in different groups (Figure 6(C)).

### 3.7. Histological analysis

**Figure 7.**
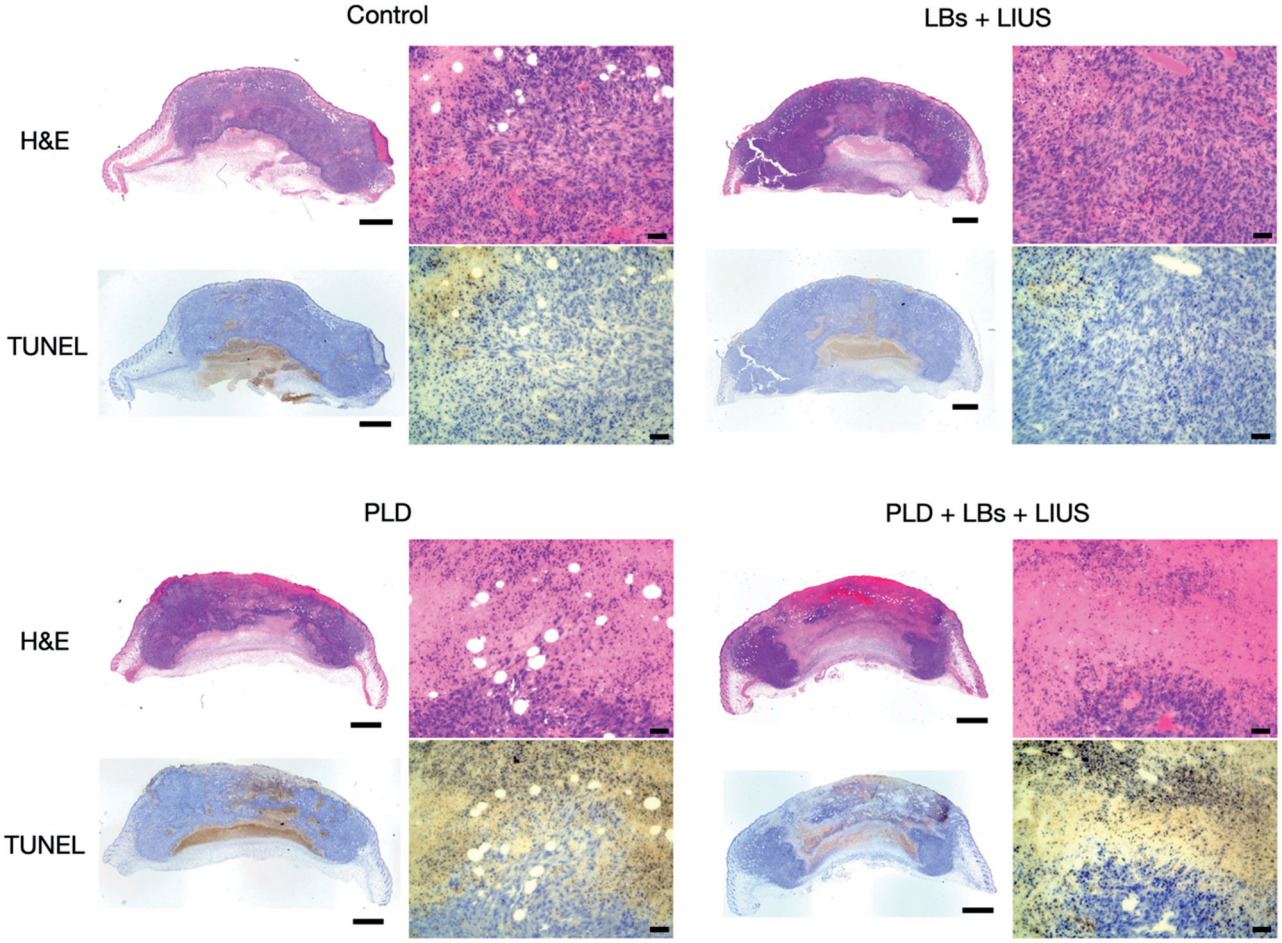
Histological examination of tumor tissues on day 8. Combination treatment with pegylated liposomal doxorubicin (PLD), lipid bubbles (LBs), and low-intensity ultrasound (LIUS) was performed on 3 days: days 0, 2, and 4. The tumors were removed on day 8, and histological examinations with hematoxylin and eosin (H&E) and terminal deoxynucleotidyltransferase-mediated dUTP nick-end labeling (TUNEL) were performed. Whole-tumor images were obtained by combining multiple low-magnification images. Scale bar: 1 mm in whole-tumor images and 50 μm in high-magnification images.

The histological images of the representative mice in the control, LBs + LIUS, PLD, and PLD + LBs + LIUS groups are shown in Figure 7. In H&E staining, the tumor tissue in the PLD group revealed moderate necrosis in the center of the lesion, while necrosis was negligible in the tumor tissues of the control and LBs + LIUS groups. Tumors treated with the combination of PLD, LBs, and LIUS showed the largest necrotic areas among the three groups. TUNEL staining revealed numerous TUNEL-positive cells around the necrotic area in the tumor tissues of the PLD and PLD + LBs + LIUS groups.

## 4. Discussion

LBs were developed for both diagnostic and therapeutic applications (Unga et al., 2019). The US contrast effect of LBs was confirmed in the kidney tissues of mice (Omata et al., 2019) and liver and tumor tissues of dogs (Yokoe et al., 2020). Previous studies have reported the promising potential of the combined use of LBs and LIUS for drug delivery (Omata et al., 2019; Unga et al., 2020; Yokoe et al., 2020). However, reports on the properties of LBs in the tumor tissues of mice are limited. Therefore, we first performed the contrast-enhanced US in tumor mice models in the present study. The injection of LBs resulted in contrast enhancement in the tumor tissue, indicating that LBs were distributed into the tumor vasculature and presented a contrast-enhancing effect. However, exposure to LIUS eliminated the contrast enhancement, suggesting that exposure to LIUS caused the destruction of LBs as a result of oscillation and inertial cavitation in the tumor vasculature.

In general, oscillation and inertial cavitation of MBs, induced by exposure to LIUS, can lead to heat and mechanical effects in a localized area (Fujishiro et al., 1998; Kondo et al., 2000). The heating effect has been utilized for hyperthermia (>43 °C)-based tumor treatment (Wust et al., 2002). We, therefore, measured tumor temperatures to evaluate whether the combined treatment using intravenous injection of LBs and exposure to LIUS led to heat generation. The temperature in tumors was increased to approximately 34 °C in the groups treated with LIUS alone and with the combination of LBs and LIUS. These results indicated that heat generation with the combined use of LBs and LIUS was not sufficient to cause hyperthermia, under the current study conditions. Therefore, it was assumed that the increase in tumor temperature by the combination of LBs and LIUS had no beneficial effect on the inhibition of tumor growth. Our results contradicted those from Suzuki et al. who previously reported that the combination of intratumoral injection of MBs and LIUS increased the tumor temperature to approximately 44 °C in mice and induced tumor growth suppression (Suzuki et al., 2016). These differences might have resulted from the administration route of MBs (intravenous vs. intratumoral injection) and the US output settings (2 W/cm^2^ for 1 min vs. 4 W/cm^2^ for 2 min).

Mechanical effect, another crucial effect induced by oscillation and inertial cavitation, is of great importance in drug delivery systems using MBs and US (Kondo et al., 2000). We aimed to elucidate the intratumoral delivery of PLD using both LBs and LIUS. The proportion of free doxorubicin and liposomal doxorubicin after the treatment was calculated *in vitro* to evaluate the influence of LBs and LIUS on the stability of PLD. The increased percentage of free doxorubicin was quite small after the treatment (1–2% larger than that of PLD without LBs and LIUS), demonstrating that the combined use of LBs and LIUS had little influence on the stability of PLD. Therefore, most of PLD particles that reached the tumor vessels might have retained their liposomal structure and pharmacokinetic features even under the combination of LBs and LIUS. *In vivo* quantitative evaluation using PLD revealed an increased intratumoral accumulation of PLD in the PLD + LBs + LIUS group. This result suggested that the combination of LBs and LIUS improved the intratumoral delivery of PLD. In addition, FITC-dextran, with a molecular weight of 70,000 Da, which was reported not to leak out from normal capillary vessels to interstitial space (Egawa et al., 2013), was used as a tracer in quantitative evaluation to detect the extravasation of macromolecular agents. Fluorescence microscopy imaging showed a wide distribution of FITC-dextran around the tumor blood vessels in the FITC-dextran + LBs + LIUS mice, indicating the promoted extravasation of the agent to the interstitial space. These findings were in accordance with those of previous reports (Unga et al., 2020; Yokoe et al., 2020) wherein increased accumulation and extravasation of macromolecular agents were observed after the combination of LBs and LIUS in normal tissues of mice. Shimizu et al. recently visualized the effect of ultrasonically excited LBs in endothelial cells, using a capillary phantom (Shimizu et al., 2020). They showed that the expansion and contraction of LBs led to the increase in local permeability through the loosening of the tight junction between endothelial cells and damage of cell membranes of the endothelial cells. Similarly, in our study, the observed results may be attributed to the increased permeability in tumor vasculature caused by oscillation and inertial cavitation of LBs. The excited LBs might mechanically open gaps or create small breaches in the tumor vessel walls, through which PLD and FITC-dextran might subsequently pass. In solid tumors, nanomedicines (20–200 nm) and macromolecular agents (>40,000 Da) tend to leak out through wide fenestrations in abnormal capillary walls and are retained due to the lack of a lymphatic drainage system (Taurin et al., 2012; Danhier, 2016). This was first conceptualized and termed as the enhanced permeability and retention (EPR) effect by Matsumura and Maeda (Matsumura & Maeda, 1986; Maeda, 2001); this concept explains the intratumoral accumulation of PLD (Barenholz, 2012). The EPR effect can be heterogeneous depending on variations in local permeability based on tumor characteristics, including impaired vascular perfusions (Hori et al., 1991), high interstitial fluid pressures (Hofmann et al., 2009), and dense stromal matrices (Yuan et al., 1994). Therefore, increasing the permeability of the tumor vasculature is an effective strategy to enhance the EPR effect, thereby improving the intratumoral accumulation of nanomedicines. Our results suggested that the combined use of LBs and LIUS enhanced the EPR effect and augmented the intratumoral delivery of PLD.

Finally, we evaluated whether the combination of LBs and LIUS could enhance the antitumor effects of PLD. In the present study, combination treatment using PLD, LBs, and LIUS exhibited strong inhibition of tumor growth with four of nine mice achieving CRs. Although there were no significant differences in tumor volume between the PLD, PLD + LIUS, and PLD + LBs + US groups at the endpoint, the former two groups had none or only one mouse showing a CR. Furthermore, H&E and TUNEL examinations showed the widest necrotic areas and a large number of apoptotic cells in the tumor tissue treated with a combination treatment. These results indicated that the antitumor effect of PLD was enhanced with the combined use of LBs and LIUS in tumor mice models. In a recent report by Bush et al., small microbubble-microdroplet clusters and LIUS were combined with an intravenous injection of PLD for the treatment of human breast cancer xenograft mice (Bush et al., 2020). The treatment significantly improved the response to treatment with PLD; however, the detailed mechanism of action was not evaluated. In the present study, the therapeutic effect might have been improved due to the increased accumulation of PLD. Ogawara et al. reported that PLD was cytotoxic to the endothelial cells of the tumor vasculature *in vivo* (Ogawara et al., 2009). Therefore, in our results, the increased accumulation of PLD might lead to not only direct cytotoxicity to the tumor cells but also indirect damage to the tumor vasculature. In clinical practice, insufficient therapeutic efficacy is a crucial factor that obstructs the wide and efficient utilization of PLD. The present study provided evidence that the combined use of LBs and LIUS was effective in enhancing the antitumor effect of PLD through localized drug delivery to the lesion.

The present study has some limitations. Our study revealed increased intratumoral delivery of PLD with the combined use of LBs and LIUS. Although the increase in vascular permeability in tumor tissues was considered an underlying effect, the detailed mechanism of action was not completely elucidated. *In vitro* vascular permeability assays are necessary to clarify the influence of the combination of LBs and LIUS on tumor endothelia. In addition, many factors were assumed to be beneficial in our system; for example, LIUS output settings (US frequency, power intensity, duty cycle, pulse repetition frequency, exposure time, etc.), the concentration of LBs, dosage of PLD, and treatment cycle. The optimal conditions were not determined due to fixed experimental settings. It is essential to identify the most crucial factor affecting our treatment technique. Moreover, a notable suppression in tumor growth was confirmed with the combination treatment in the tumor growth inhibition test, while no significant differences in tumor volume were found between treatment with PLD alone and in the combination treatment at the endpoint. Further *in vivo* investigations under additional experimental conditions should be conducted to elucidate the optimal experimental settings and true therapeutic benefits associated with the combination treatment of PLD, LBs, and LIUS.

## 5. Conclusions

The present study aimed to elucidate the underlying effects and detailed therapeutic benefits of the combined treatment using PLD, LBs, and LIUS in tumor mice models. The combined use of LBs and LIUS improved the intratumoral accumulation of PLD. Furthermore, combination treatment with PLD, LBs, and LIUS strongly inhibited tumor growth, suggesting that combined LBs and LIUS enhanced the antitumor effects of PLD through increased intratumoral delivery. Although further studies are required to clarify the detailed mechanism of the improved intratumoral accumulation of PLD, the present study provided evidence that the combination of LBs and LIUS was successful in effectively delivering PLD for tumor treatment. This combination is highly promising for the delivery of other macromolecular agents such as DNA and antibody drugs.
